# Experience with arthroscopic treatment of disorders in the sternoclavicular joint: A prospective series of 78 patients

**DOI:** 10.1002/ksa.70298

**Published:** 2026-01-26

**Authors:** Anna Hoerby Normann Rasmussen, Martin Wyman Rathcke, Michael Rindom Krogsgaard

**Affiliations:** ^1^ Section for Sports Traumatology M51, Department of Orthopedic Surgery Copenhagen University Hospital Bispebjerg Copenhagen Denmark; ^2^ Institute for Clinical Medicine, Copenhagen University Copenhagen Denmark

**Keywords:** arthroscopy, disk resection, open surgery, osteoarthritis, sternoclavicular joint

## Abstract

**Purpose:**

To evaluate pain, function and patient‐reported outcomes following arthroscopically intended treatment of painful sternoclavicular joint (SCJ) conditions between 2010 and 2024 in a consecutive cohort with long‐term follow‐up including 78 patients. We hypothesised that this procedure would reduce pain and improve function, even when conversion to open surgery was required.

**Methods:**

Seventy‐eight patients with SCJ pain unresponsive to ≥12 months of conservative therapy were scheduled for arthroscopic surgery. Outcomes were assessed using the Disabilities of the Arm, Shoulder and Hand (DASH) score and the Oxford Shoulder Score (OSS). Follow‐up included assessments at 1, 2 and 5 years, as well as a final cross‐sectional evaluation (mean 7.2 years), capturing function, work ability, sports participation and satisfaction.

**Results:**

In 24 patients, the procedure was converted to open surgery, primarily due to obstructing osteophytes, insufficient visualisation, or narrow joint space. Sixty‐four patients (82%) completed long‐term follow‐up. Significant improvements were observed in DASH (61.8 ± 19.2 to 16.1 ± 18.0; *p* < 0.00001) and OSS pain sub‐scores for worst pain (2.65 ± 0.9 to 1.1 ± 1.0; *p* < 0.00001). Five patients (6%) underwent reoperation. Sports‐related limitations improved from 75.9% preoperatively to 15.6% postoperatively. At final follow‐up, 87% reported complete or partial symptom resolution, and 90% would choose surgery again.

**Conclusions:**

Arthroscopically intended SCJ treatment provides significant and sustained improvements in pain and function. Although conversion to open surgery was required in approximately one‐third of cases, overall outcomes were favourable, with high satisfaction and low complication and reoperation rates.

**Level of Evidence:**

Level IV.

**Abbreviation**
SCJ: sternoclavicular joint

## INTRODUCTION

The sternoclavicular joint (SCJ) is a key articulation connecting the upper extremity to the axial skeleton and transmitting considerable load through the shoulder girdle. Although degenerative changes of the SCJ are frequently observed in older individuals, only a minority become symptomatic [12, 17]. Patients with symptomatic SCJ conditions typically report localised pain, tenderness, swelling, and radiation of discomfort toward the neck, chest, or shoulder, often limiting daily activities, work, and sports participation [4, 6, 19].

Conservative treatment – including load modification, analgesics, corticosteroid injections, and physiotherapy – is generally recommended as first‐line management, but some patients continue to experience substantial pain and disability despite prolonged non‐operative care [19, 22]. In such cases, surgical intervention may be considered.

Evidence for operative management of SCJ pathology remains limited, with most published studies reporting small cohorts treated either by open or arthroscopic techniques [1, 3, 5, 10, 14, 15, 20, 21, 22, 23, 24, 25, 26, 27]. Concerns about the proximity of mediastinal structures, in particular vessels and lungs, have contributed to the historically cautious approach to SCJ surgery [2, 28]. Arthroscopy has been described by only a few centres, and the existing arthroscopic series originate largely from a single institution [24, 25, 26], leaving a need for larger, prospectively collected datasets from additional centres.

Arthroscopy offers potential advantages, such as improved visualisation of intra‐articular pathology, reduced soft‐tissue morbidity, and the option to convert to open surgery when necessary [9, 11, 18]. However, long‐term outcomes following arthroscopically intended SCJ treatment and the clinical implications of conversion remain insufficiently documented.

We hypothesised that arthroscopically intended treatment of the SCJ –including debridement and resection of a pathological disc or osteophytes – would reduce pain and improve function without increasing complication rates. The aim of this study was therefore to evaluate pain, functional outcomes, and patient satisfaction in a large, consecutive cohort undergoing arthroscopically intended treatment for painful SCJ conditions and to report our experience regarding indications, feasibility, and the need for conversion to open surgery.

## METHODS, PREOPERATIVE DIAGNOSIS AND TREATMENT OPTIONS

This prospective cohort study included consecutive patients who underwent arthroscopically intended treatment for painful sternoclavicular joint (SCJ) conditions at Copenhagen University Hospital Bispebjerg between 2010 and 2024. Patients from all regions of Denmark were referred for evaluation.

Inclusion criteria were as follows:

1. SCJ pain for ≥12 months,
2. failure of conservative treatment, including load modification, physiotherapy and corticosteroid injection, and
3. imaging findings consistent with symptomatic SCJ pathology, including osteoarthritis, disc degeneration or rupture, osteophytes, synovitis or osteochondral changes.

Exclusion criteria included age <16 years, SCJ instability, previous SCJ surgery, acute or chronic infection, post‐infectious sequelae, fractures and inflammatory arthropathy requiring specialised rheumatologic management.

**Figure 1:**
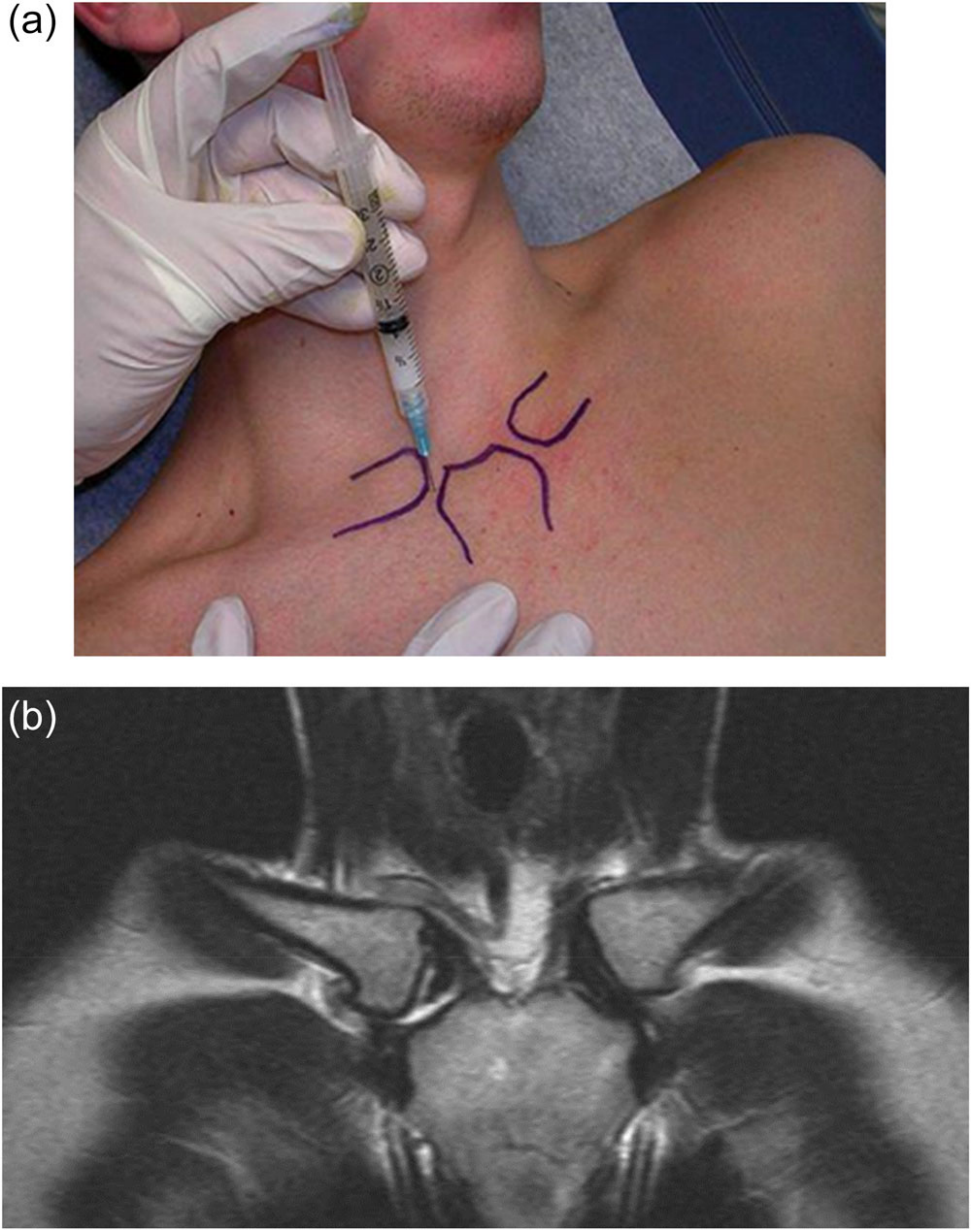
(a) Corticosteroid injection in the SCJ. (b) Pre‐operative scan demonstrating right SCJ osteoarthritis and rupture of the articular disc.

All patients underwent standardised clinical evaluation, including palpation, assessment of swelling, pain provocation during arm elevation or horizontal adduction and evaluation for referred pain to the neck or shoulder. Imaging consisted of computed tomography (CT) to assess joint degeneration and osteophyte formation, and magnetic resonance imaging (MRI) to evaluate soft‐tissue structures including the intra‐articular disc (Figure 1b). All scans were interpreted by an experienced radiologist in collaboration with the operating SCJ surgeon.

To confirm the SCJ as the primary pain generator, all patients received an ultrasound‐guided diagnostic injection of local anaesthetic (Figure 1a). Patients with a clear but temporary reduction in symptoms were considered candidates for surgery.

### Surgical technique

All procedures were performed using a standardised arthroscopic technique as previously described [11, 18]. Before surgery, the patient′s blood type was registered and blood was made available as a precaution, although severe bleeding during SCJ arthroscopy has not been reported in the literature [16]. Procedures were conducted under general anaesthesia with the patient in a supine position, the scapula supported, and the head rotated contralaterally to optimise access to the joint.

**Figure 2:**
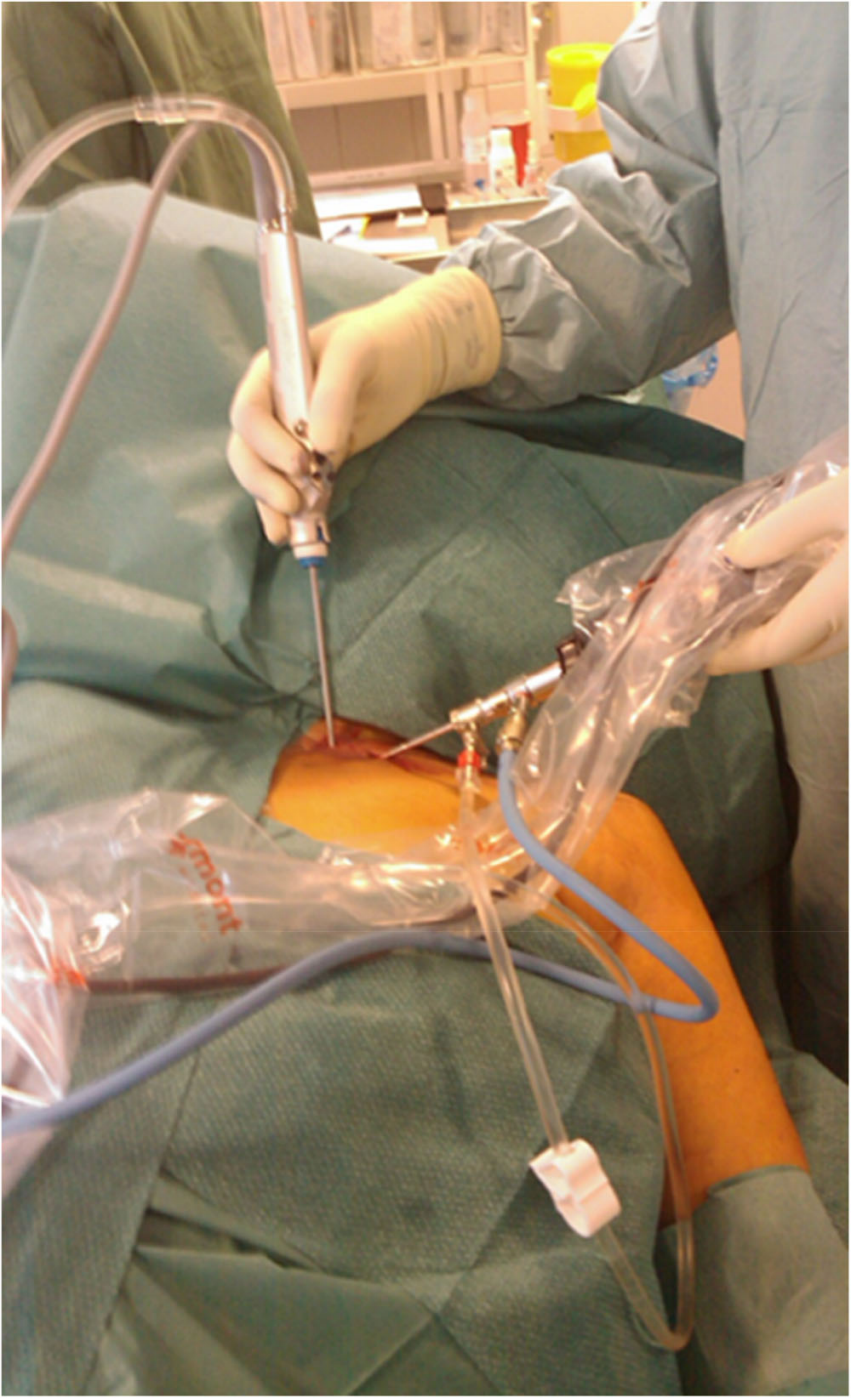
Illustration of the surgical setup.

**Figure 3:**
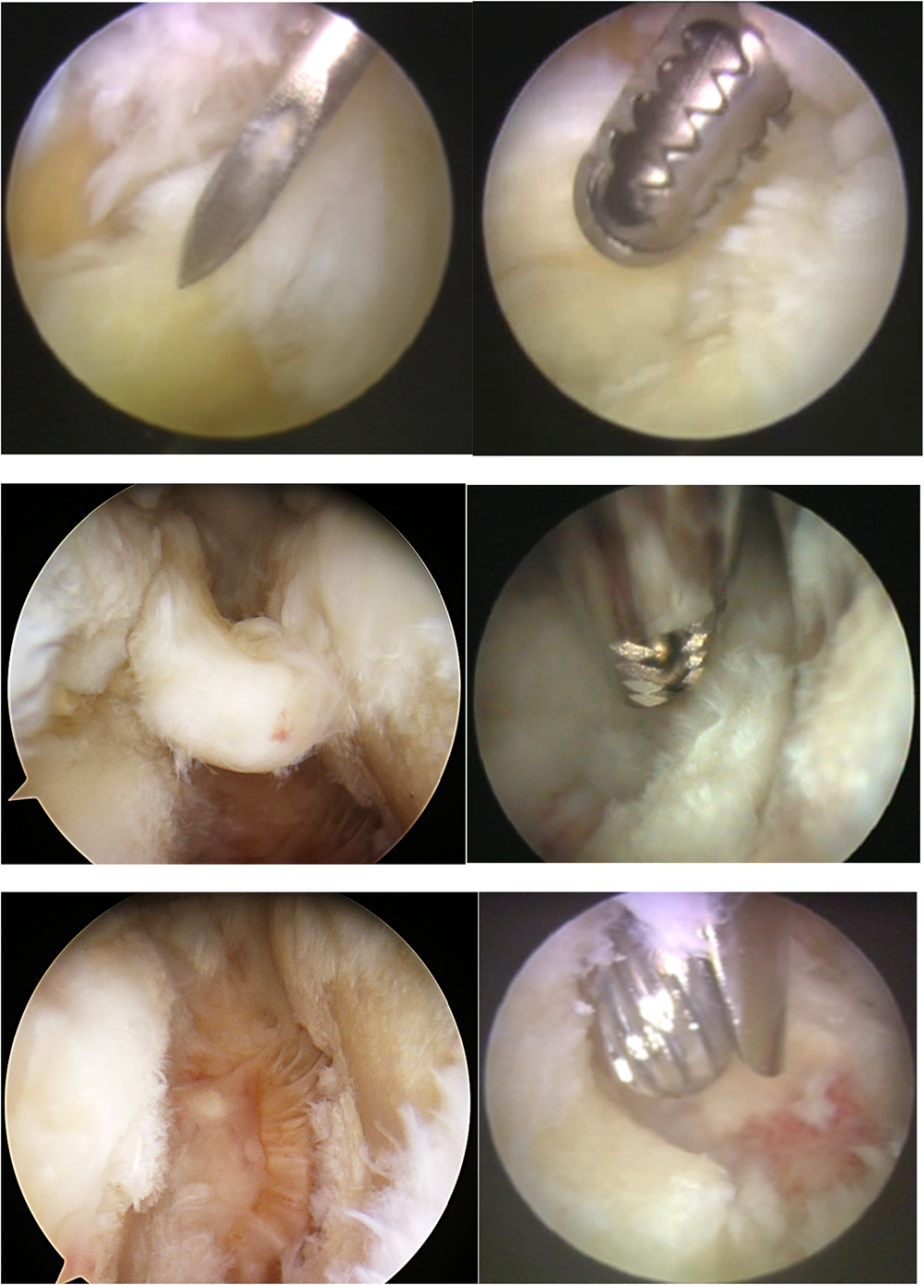
Images illustrating the arthroscopic procedure. Top‐left: second portal identified with a needle. Top‐right: shaver inserted. Middle‐left: rupture of the articular disc. Middle‐right: Resection of the torn disc. Bottom‐left: posterior capsule of SCJ after resection of the torn disc. Cartilage changes grade IV on the manubrium (right) and grade 2‐3 on the clavicle (left). Bottom‐right: resection of the medial clavicular end.

Two portals were established: an inferior portal for the arthroscope and a superior portal for instrumentation, guided by direct visualisation to avoid posterior capsular violation [11, 23] (Figures 2 and 3). Joint distension with saline confirmed intra‐articular access. The entire joint was systematically inspected, and pathology was treated by synovectomy, debridement of the degenerative disc, removal of loose bodies and osteophytes, and resection of the medial clavicle end when indicated.

Conversion to open surgery was performed when arthroscopic progression was not feasible due to (1) obstruction from large extra‐articular osteophytes, (2) insufficient visualisation, (3) a narrow joint space, or (4) the need for disc repair or reattachment that could not be performed arthroscopically. The conversion criteria were predefined, based on previously published anatomical and technical considerations [2, 11, 13, 18, 28], and implemented consistently throughout all operations.

### Postoperative care and rehabilitation

All procedures were performed as outpatient surgery. When disc repair was undertaken, a fixed sling was used for 2 weeks; otherwise, a soft sling was used for comfort only, and the arm was mobilised as tolerated. All patients received standardised instructions and were referred to a physiotherapist with experience in SCJ rehabilitation, with the option of a structured municipal physiotherapy programme. This approach is consistent with published rehabilitation recommendations following SCJ procedures [5, 10].

### Patient outcomes

Patient‐reported outcome measures (PROMs) were collected preoperatively and at 1‐, 2‐ and 5‐year follow‐up using the Disabilities of the Arm, Shoulder and Hand (DASH) score and the Oxford Shoulder Score (OSS). The primary outcome was pain as measured by the four questions in the OSS: ‘During the last 4 weeks: How would you describe the worst pain in your shoulder? How would you describe the normal pain level in your shoulder? How much have your shoulder pain disturbed your ability to work?’, and ‘How would you describe the pain in your shoulder during the night?’

Because no SCJ‐specific PROM exists [7, 8], DASH and OSS were selected for their established use in shoulder‐related conditions and their ability to detect functional change.

OSS pain sub‐items were analysed separately to capture SCJ‐related pain during daily activities, recognising that this use has not been specifically validated for SCJ pathology but offers clinically meaningful resolution of pain domains in the absence of a joint‐specific score [7].

At the final cross‐sectional follow‐up in June 2024, additional data were collected on work ability, sports participation, satisfaction, complications, and reoperations.

The ethical committee for the Capital Region stated that ethical permission for the current study was not necessary, as there was no intervention, and permission to contact the patients and store data was obtained from the data authorities. Before surgery, all patients had signed a consent form to allow us to contact them and for their data to be anonymously used for research purposes.

### Statistical analysis

Descriptive statistics using Microsoft Excel were used to summarise demographic data, diagnoses, procedures, and outcomes. Continuous variables were reported as means with standard deviations, categorical variables as counts and percentages. Comparative analyses across follow‐up time points and between arthroscopic and converted procedures used simple comparative methods. A *p*‐value of <0.05 was considered statistically significant, consistent with prior SCJ outcome studies [5, 10, 15].

## RESULTS

### Demographics

A total of 78 patients (51 women, 27 men; mean age 45.9 years, range 16–79) underwent arthroscopically intended surgery for SCJ‐related pain. The right SCJ was affected in 64% of cases, which was significantly more frequent than the left (*p* < 0.05). No bilateral procedures were performed.

Sixty‐four patients (82%) completed long‐term follow‐up; 3 patients died of unrelated causes, and 11 were lost to follow‐up. The mean follow‐up duration for respondents was 7.2 years (range 1–14). Most patients had multiple diagnoses (up to three), as detailed in Table 1, and underwent more than one procedure (Table 2).

**Table 1:** Diagnoses of the 78 consecutive patients scheduled for arthroscopic treatment for pain in the SCJ.

Diagnosis	*N*
Osteoarthritis	52
Disc rupture	53
Loose bodies	7
Synovitis	27
Focal osteochondral changes	20
Extraarticular exostoses	6

Abbreviation: SCJ, sternoclavicular joint.

**Table 2:** Treatments for the 78 consecutive patients scheduled for arthroscopic treatment for pain in the SCJ.

Treatment	*N*
Resection of disc	49
Cartilage debridement	35
Resection of medial clavicle end	26
Resection of osteophytes	13
Removal of loose bodies	4
Synovectomy	18

Abbreviation: SCJ, sternoclavicular joint.

### Surgical procedures and conversion to open surgery

All procedures were initiated arthroscopically. Conversion to open surgery was required in 24 patients (31%) due to obstructing osteophytes (*n* = 9), insufficient visualisation (*n* = 8), narrow joint space (*n* = 4), or the need for disc repair (*n* = 3), as summarised in Table 3. Among converted cases, 17 patients completed long‐term follow‐up.

**Table 3:** Indication for conversion to open surgery in 24 of the 78 consecutive patients scheduled for arthroscopic treatment for pain in the SCJ.

Indication	*N*
Insufficient overview of the joint	8
Access blocked by extraarticular osteophytes	9
Narrow joint	4
Repair/re‐attachment of the disc	3

Abbreviation: SCJ, sternoclavicular joint.

Outcomes for patients who underwent conversion did not differ significantly from those treated arthroscopically, as shown in Table 4.

**Table 4:** Comparison of results from patients treated arthroscopically vs. patients in whom the arthroscopic procedure was converted to open (both after 7.2 years).

Score	Converted to open (17 patients)	7.2 years post‐op. (47 patients)
DASH	15.2	16.5
DASH work	33.4	28.3
Worst pain (OSS)	1.0	1.1
Usual pain (OSS)	1.0	1.2
Pain during work (OSS)	0.8	1.1
Pain at night (OSS)	0.9	1.4

*Note*: There was no significant difference between the groups (*p* > 0.05).

Abbreviations: DASH, Disabilities of the Arm, Shoulder and Hand; OSS, Oxford Shoulder Score.

### Functional outcomes

Substantial improvements were observed across all functional measures (Table 5). The mean DASH score improved from 61.8 (SD 19.2) preoperatively to 16.1 (SD 18.0) at final follow‐up (*p* < 0.00001). Similar significant improvements were observed in OSS pain sub‐scores: worst pain decreased from 2.65 (SD 0.9) to 1.1 (SD 1.0); usual pain from 2.3 (SD 0.8) to 1.1 (SD 0.9); work‐related pain from 2.1 (SD 0.9) to 1.0 (SD 0.9); and night pain from 2.5 (SD 1.1) to 1.3 (SD 1.4).

**Table 5:** Functional outcomes for patients scheduled for arthroscopic treatment for pain in the SCJ.

Score	Pre‐op. (49 patients)	1 year post‐op. (38 patients)	2 years post‐op. (25 patients)	5 years post‐op. (14 patients)	7.2 years post‐op. (64 patients)	Mean improvement
DASH	61.8 (SD: 19.2)	48.5 (SD: 19.5)	50.9 (SD: 23.8)	39.9 (SD: 12.7)	16.1^a^ (SD: 18.0)	73.9%
DASH work	70.8 (SD: 42.4)	51.4 (SD: 30.0)	51.4 (SD: 29.4)	39.7 (SD: 13.0)	29.2^b^ (SD: 31.8)	58.7%
Worst pain (OSS)	2.65 (SD: 0.9)	1.9 (SD: 0.9)	1.8 (SD: 1.2)	1.4 (SD: 0.8)	1.1^a^ (SD: 1.0)	58.5%
Usual pain (OSS)	2.3 (SD: 0.8)	1.5 (SD: 1.0)	1.2 (SD: 1.0)	1.1 (SD: 0.5)	1.1^a^ (SD: 0.9)	52.2%
Pain during work (OSS)	2.1 (SD: 0.9)	1.4 (SD: 1.1)	1.4 (SD: 1.3)	1.0 (SD: 0.9)	1.0^a^ (SD: 0.9)	52.4%
Pain at night (OSS)	2.5 (SD: 1.1)	1,6 (SD: 1.2)	1.4 (SD: 1.5)	1.3 (SD: 1.1)	1.3^b^ (SD: 1.4)	48%

*Note*: For changes from preoperatively to 7.2 years postoperatively: a = *P* < 0.00001, b = *P* < 0.0001.

^a^ *p* < 0.00001.

^b^ *p* < 0.0001.

Functional gains were consistent across 1‐, 2‐ and 5‐year follow‐up intervals (Table 4), and no deterioration was observed over time.

### Work‐related outcomes

Preoperatively, 70.8% of employed patients reported pain‐related work limitations. At final follow‐up, only 2.6% reported persistent work‐related limitations due to SCJ pain. Overall, 93% of those employed preoperatively remained employed at follow‐up (Table 6).

**Table 6:** Statements from patients operated for pain in the SCJ in relation to work.

Question	Yes	Sometimes	No
If you worked before the surgery: Did the pain from your SCJ give you trouble in performing your work?	70.8% (29)	19.5% (8)	9.7% (4)
Do you work now?	59.4% (38)	Not relevant	40.6% (26)
If you work now: Do you have trouble performing your work due to pain from your SCJ?	2.6% (1)	42% (16)	55.4% (21)

Abbreviation: SCJ, sternoclavicular joint.

### Sports participation

Before surgery, 75.9% of patients reported limitations in sports participation due to SCJ pain. After treatment, this proportion had decreased to 15.6% (Table 7). Among patients who had previously stopped sports because of SCJ symptoms, 47% resumed sports participation after surgery.

**Table 7:** Statements from patients operated for pain in the SCJ in relation to performing sports.

Question	Yes	Sometimes	No
If you performed sports before the surgery: Did the pain from your SCJ give you trouble in performing sports?	75,9% (22)	20,7% (6)	3,4% (1)
If you did not perform sport before your surgery: Was the pain from your SCJ the reason?	43% (15)	Not relevant	57% (20)
If yes to the question above: Is it now (after your surgery) possible for you to perform sport as you wish?	42,8% (7)	Not relevant	57,2% (8)
Do you now have trouble performing sports due to pain from your SCJ?	15,6% (10)	37,5% (24)	46,9% (30)

Abbreviation: SCJ, sternoclavicular joint.

### Complications and reoperations

No major complications such as bleeding, infection, pneumothorax, or injury to mediastinal structures were observed. Five patients (6%) underwent reoperation: two required additional osteophyte removal, two had further medial clavicle resection, and one had scar tissue excised.

### Patient satisfaction

At final follow‐up, 87% of patients reported that their symptoms had been completely or partially resolved, and 90% would choose surgery again. Details are shown in Table 8.

**Table 8:** Statements from patients operated for pain in the SCJ in relation to satisfaction with surgery.

Did the surgery solve the problem in your SCJ?	
Yes, completely	36%
Yes, partially	51%
No, the problem is the same as before the surgery	10%
No, the problem has become worse after the surgery	3%
If the problem was completely or partially solved after the surgery: Has the problem returned?	
No, it has not changed	89,2%
Yes, I have gained more problems with time	10,8%
Are you satisfied with the result of the surgery?	
Yes	77%
Neither/nor	18%
No	5%
Would you have chosen the surgery, if you beforehand had known the outcome of treatment?	
Yes	90%
Maybe	6,7%
No	3,3%
Will you choose surgery, if you experience the same problem in the other SCJ?	
Yes	76,8%
Maybe	16,7%
No	6,7%

Abbreviation: SCJ, sternoclavicular joint.

## DISCUSSION

The main finding of this study was that arthroscopically intended treatment of painful SCJ conditions resulted in substantial and sustained improvements in pain, function and patient satisfaction. These improvements were consistent across all follow‐up intervals up to a mean of 7.2 years and were accompanied by a low complication rate and a modest reoperation rate of 6%. Importantly, outcomes did not differ significantly between patients treated arthroscopically and those in whom conversion to open surgery was necessary.

The present findings align with previous reports demonstrating favourable results after operative treatment of SCJ osteoarthritis, both through open resection arthroplasty [1, 3, 5, 10, 14, 15, 20, 21] and arthroscopic approaches [24, 25, 26]. Our results expand on these studies by providing long‐term data from the largest prospectively followed cohort undergoing arthroscopically intended SCJ treatment to date. Similar to Dekker et al. [5], who reported an 86% return‐to‐sport rate following open medial clavicle resection, we observed a marked reduction in sport‐related limitations, indicating that surgical management – whether arthroscopic or open – can meaningfully restore shoulder girdle function.

### Conversion to open surgery

A notable finding was the conversion rate of 31%. Previous arthroscopic series have not reported conversion rates [24, 25, 26], possibly due to preoperative patient selection or exclusion of converted cases. In our cohort, conversions were primarily required due to obstructing osteophytes, insufficient visualisation, or very narrow joint spaces. These indications are consistent with published technical and anatomical descriptions highlighting the challenges of arthroscopic access to the SCJ [2, 11, 18, 28]. Importantly, patients who underwent conversion achieved outcomes comparable to those treated fully arthroscopically, suggesting that conversion is not a negative prognostic factor when appropriately indicated.

### Functional outcomes

Functional improvements were substantial, with DASH scores improving from ‘severe disability’ to ‘mild or no disability’, consistent with prior work on surgical management of SCJ disorders [3, 5, 10]. Pain reduction across OSS sub‐items further supported meaningful symptomatic relief. Because no SCJ‐specific patient‐reported outcome measure exists [7, 8], DASH and OSS were selected to provide validated assessment of upper‐limb function and pain. The consistent improvement across multiple follow‐up intervals suggests that surgical benefit is valid and does not deteriorate over time.

### Return to work and sports

Return to work was high, with only 2.6% reporting persistent work‐related limitations at long‐term follow‐up. Sports participation also improved markedly, with nearly half of previously inactive patients resuming sports after treatment. These results mirror those of Dekker et al. [5] and support the clinical relevance of operative treatment in restoring high‐demand physical function.

### Safety and complications

No major complications, including mediastinal injury, pneumothorax, or infection, were observed. This aligns with prior literature demonstrating that, despite proximity to critical mediastinal structures, SCJ surgery can be performed safely with appropriate technique and surgeon experience [2, 28]. The low reoperation rate further supports the long‐term durability of this treatment.

### Limitations

This study has several limitations. First, it is a single‐centre cohort, which may limit generalisability, although consistent technique and follow‐up strengthen internal validity. Second, the absence of a control group introduces a risk of overestimating treatment effects. Third, loss to follow‐up occurred despite multiple contact attempts, although the final follow‐up rate of 82% is acceptable for long‐term surgical studies. Fourth, DASH and OSS are not developed or validated for SCJ disorders [7, 8], and a joint‐specific PROM would provide a more precise assessment.

## CONCLUSION

Arthroscopically intended treatment of painful sternoclavicular joint conditions resulted in significant and sustained improvements in pain, function, and patient satisfaction at long‐term follow‐up. Complication and reoperation rates were low. One‐third of procedures required conversion to open surgery, but outcomes were comparable between arthroscopic and converted cases. These findings support arthroscopically intended treatment as an effective surgical option for selected patients with persistent SCJ symptoms following failed conservative management.

## AUTHOR CONTRIBUTIONS

*Data collection, analysis and manuscript drafting*: Anna H. N. Rasmussen. *Surgical procedures and data interpretation*: Martin Wyman Rathcke. *Surgical procedures, supervision and manuscript revision*: Michael R. Krogsgaard.

## CONFLICT OF INTEREST STATEMENT

The authors, their immediate family and any research foundation with which they are affiliated did not receive any financial payments or other benefits from any commercial entity related to the subject of this article.

## ETHICS STATEMENT

The ethical committee for the Capital Region of Denmark declared that ethical permission for this study was not necessary, as there was no intervention. Decision number: F‐24005033. All patients provided informed consent for participation.

## Data Availability

The data that support the findings of this study are not publicly available due to privacy/ethical restrictions; they are available from the corresponding author upon reasonable request.
